# Effect of hydro-alcoholic extract of *Nigella sativa* on cisplatin-induced memory impairment and brain oxidative stress status in male rats

**DOI:** 10.22038/AJP.2023.22789

**Published:** 2024

**Authors:** Yasin Mahmoud Janloo, Fatemeh Sadat Attari, Sahar Roshan, Hadi Lotfi, Amir Hossein Pezeshki, Masoud Hosseinzadeh, Batool Shakiba-Jam, Marzieh Kafami

**Affiliations:** 1 *Student Research Center, Sabzevar University of Medical Sciences, Sabzevar, Iran *; 2 *Leishmaniasis Research Center, Sabzevar University of Medical Sciences, Sabzevar, Iran *; 3 *Department of Pathology, School of Medicine, Isfahan University of Medical Sciences, Isfahan, Iran*; 4 *Non-Communicable Disease Research Center, Sabzevar University of Medical Sciences, Sabzevar, Iran*; 5 *Department of Physiology and Pharmacology, Faculty of Medicine, Sabzevar University of Medical Sciences, Sabzevar, Iran*

**Keywords:** Nigella sativa, Cisplatin, Memory, Malondialdehyde, Superoxide dismutase, Thiol

## Abstract

**Objective:**

Studies have shown the complications of chemotherapy on learning and memory. Empirical evidence suggests that *Nigella sativa* (NS) has neuroprotective activities. Therefore, the aim of our study was to investigate the effects of NS on cisplatin-induced memory impairment.

**Materials and Methods:**

This study was conducted on 40 male rats grouped as: control (saline: 2 ml/kg, intraperitoneally (IP), once weekly/2 weeks), cisplatin (Cis, 2 mg/kg, IP, once weekly/2 weeks), NS (200 mg/kg, IP, once weekly/2 weeks), Cis +NS 200 (2 mg/kg Cis + 200 mg/kg NS, IP, once weekly/2 weeks), and Cis +NS 400 (2 mg/kg Cis + 400 mg/kg NS, IP, once weekly/2 weeks). Morris water maze (MWM) test was used to assess spatial learning and memory. In addition, superoxide dismutase (SOD) activity, and thiol and malondialdehyde (MDA) levels were evaluated in the brain.

**Results:**

Cis significantly enhanced the traveled distance and time spent in the target quadrant in the MWM test. Additionally, MDA levels increased in the Cis group, while thiol and SOD decreased in this group. As a result of treatment with NS, behavioral results were reversed in the groups receiving NS compared to the Cis group. Also, NS reduced MDA level but improved SOD and thiol levels in brain tissue samples.

**Conclusion:**

NS could improve memory impairment and oxidative stress in animals receiving Cis. Therefore, NS could be used as a potential food supplement to prevent neurotoxicity in patients undergoing chemotherapy.

## Introduction

Platinum-based chemotherapy is widely used to treat different cancers. Cisplatin is the foremost agent in platinum drugs for treating ovarian, bladder, and testicular tumors (Cui et al., 2000; Dasari et al., 2014). However, the side effects of cisplatin such as nephrotoxicity and hepatotoxicity restrict its clinical applications in oncology (Zicca et al., 2002; Rostami et al., 2014; Nematbakhsh et al., 2017). Previous researches have confirmed the toxicity of the drug on the nervous system as a significant side-effect (Robbins et al., 2002; Amptoulach et al., 2011; Hosseinzadeh et al., 2021). Moreover, cognitive deficits such as memory and attention difficulties have been experienced by the patients undergoing chemotherapy (Robbins et al., 2002; Ahles et al., 2007; Vardy et al., 2007). Cisplatin can lead to neurotoxic effects in the nervous system due to both oxidative damages and activation of inflammatory factors (Jaggi et al., 2012).

Therefore, finding a safe adjunct therapy to prevent cisplatin-induced neurotoxicity is an essential objective in preclinical studies. 

The health effects of traditional medicines or foods have attracted attention in recent decades. *Nigella sativa* (NS) is a protective agent which has been utilized increasingly to manage various diseases (Seghatoleslam et al., 2016). NS contains thymoquinone, anthocyanins, flavonoids, alkaloids, and essential fatty acids. Furthermore, NS phytochemicals are primarily responsible for its beneficial effects on diseases. The NS has been used for treating many diseases including headaches, pain, hypertension and gastrointestinal illnesses (Badreldin et al., 2003; Boskabady et al., 2005; Boskabady et al., 2007; Boskabady et al., 2008).

According to the literature, NS is significantly protective against neural damage and it improves memory and cognition (Bin Sayeed et al., 2010). The antioxidant attributes of NS have reported during cerebral ischemia in experimental tests (Hosseinzadeh et al., 2007). Some studies have also indicated that NS antioxidant properties have neuroprotective effects on cognitive impairment (Khan et al., 2008; Sahak et al., 2013; Sahak et al., 2016; Azzubaidi et al., 2011).

The main aim of our work was to evaluate the effects of NS on cisplatin-induced memory deficit in male rats.

## Materials and Methods

**Preparation of the herbal extracts**

First, plants were collected from Khorasan, Iran. NS seeds were crushed, made into flour and extracted using a Soxhlet device with ethanol. Then the solution was concentrated by using compression process. Finally, it was dissolved in saline normal before administration (Hosseini et al., 2015). 

**Animal handling and housing**

A total of 40 Wistar male rats weighing 250-270 grams, aged eight weeks, were housed under standard temperature (23-25°C) with 12 hr light: 12 hr dark cycle. Experiments were done in accordance with the Ethics Committee Guidelines for Research on Laboratory Animals of Sabzevar University of Medical Sciences (IR.MEDSAB.RES. 1398.084).

The behavioral tests were performed simultaneously during the day.

**Grouping and treatments**

The animals were classified into five groups (n=8), including control (saline: 2 ml/kg, once weekly/2 weeks), Cis (5 mg/kg of cisplatin intraperitoneal (IP), once weekly/2 weeks), NS (200 mg/kg, IP, once weekly /2 weeks); Cis + NS 200 (2 mg/kg Cis + 200 mg/kg NS, IP, once weekly/2 weeks), and Cis + NS 400 (2 mg/kg Cis + 400 mg/kg NS, IP, once weekly/2 weeks). The behavioral test was performed on six consecutive days.

**MWM apparatus and procedures**

The Morris water maze (MWM) test was conducted in order to evaluate the influences of NS and cisplatin on memory and learning. MWM is a circular apparatus with a diameter of 140 cm and a depth of 55 cm. In this study, the round pool was filled with water at the temperature of 23-24C. The southwest quadrant of the tank also had a round platform 2 cm below the water surface. Four trials (north, south, east and west) were performed for six consecutive days. The acquisition test was conducted for five consecutive days, followed by a probe test on the sixth day. 

A camera was placed over the pool to record the animals' swimming paths during the examination sessions. A tracking software was used to calculate the escape latency to reach the hidden platform. The time of swimming was 60 sec. On the prob test (the sixth day), the animals' ability to remember the platform location was assessed through calculating the traveled distance and time spent in the target quadrant (Vafaee et al., 2015).

**Biochemical assessment**

Rats were euthanized with the over dose of urethane. After removing the brain of animals, hippocampus and cortex were isolated. The tissue samples were placed in microtubes and stored at -80C. The brain tissue level of malondialdehyde (MDA) was evaluated to measure lipid peroxidation. A homogenate of brain tissue was treated with thiobarbituric acid, trichloroacetic acid, and HCl. Next, they were incubated in a 100ºC water bath (40 min), and the supernatants were obtained after centrifugation at 1000 rpm (10 min). The absorbance values read at 535 nanometers (Shakiba-Jam et al., 2021).

At the next stage, the total thiol content was measured using 1,000 µl of ethylenediaminetetraacetic acid-Tris buffer, which was added to 50 µl of samples. Then, 20 µl of 2, 2′-dinitro-5, and 5′dithiodibenzoic acid solution (10 mM) was added and the solution was kept at room for at least 15 min. The absorbance was measured at 412 nanometers (Shakiba-Jam et al., 2021; Hosseinzadeh et al., 2022). The Madesh protocol was used to determine superoxide dismutase (SOD) activity (Madesh et al., 1998). The enzyme activity was read at 570 nm.

**Statistical analysis**

Obtained data is presented as means±SEM. The repeated-measure test followed by Tukey post-hoc was used to analyze escape latency during five days of MWM. The data of the biochemical test, and percentage of time and traveled distance in target quadrant were analyzed by using One-way ANOVA followed by Tukey post-hoc test. 

## Results

**Effects of cisplatin and NS on learning and memory**

**Figure 1:**
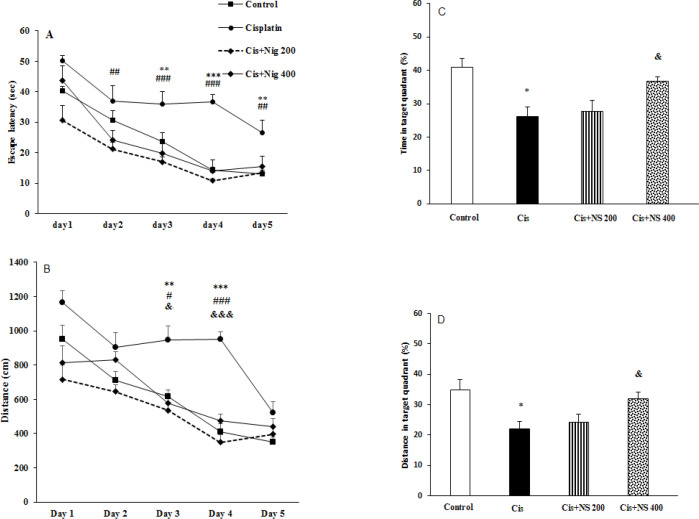
Comparison of A) Time Latency, B) Traveled distance, C) Percentage of time spent in the target quadrant and D) Percentage of traveled distance in the target quadrant in MWM Test among the groups. Data is expressed as mean±SEM; ***p<0.001 and **p<0.01 compared to the Cis and control groups; ###p<0.001, ##p<0.01 and #p<0.05 compared to Cis + NS 200 and Cis groups; &&&p<0.001, &&p<0.01 and &p<0.05 compered to Cis + NS 400 and Cis groups [n=8].

Behavioral tests were performed using the MWM after completing cisplatin treatment. Compared to the control group, the escape latency in the Cis group was significantly enhanced (p<0.01 and p<0.001) (Figure 1-A). In addition, the animals of the Cis group had shorter time and traveled distance in the target quadrant compared to the control group (p<0.05 and p<0.05, Figure 1-B and 1-C). 

Receiving both doses of NS decreased the mean latency time compared to the Cis group (p<0.01 on the second and fifth days, p<0.001 on the third and fourth days, Figure 1-A). On the other hand, the administration of NS enhanced the traveled distance and spent time in the target quadrant compared to the Cis group (p<0.05, Figures 1-B and 1-C). 

**Effects of cisplatin and NS on brain MDA levels**

**Figure 2:**
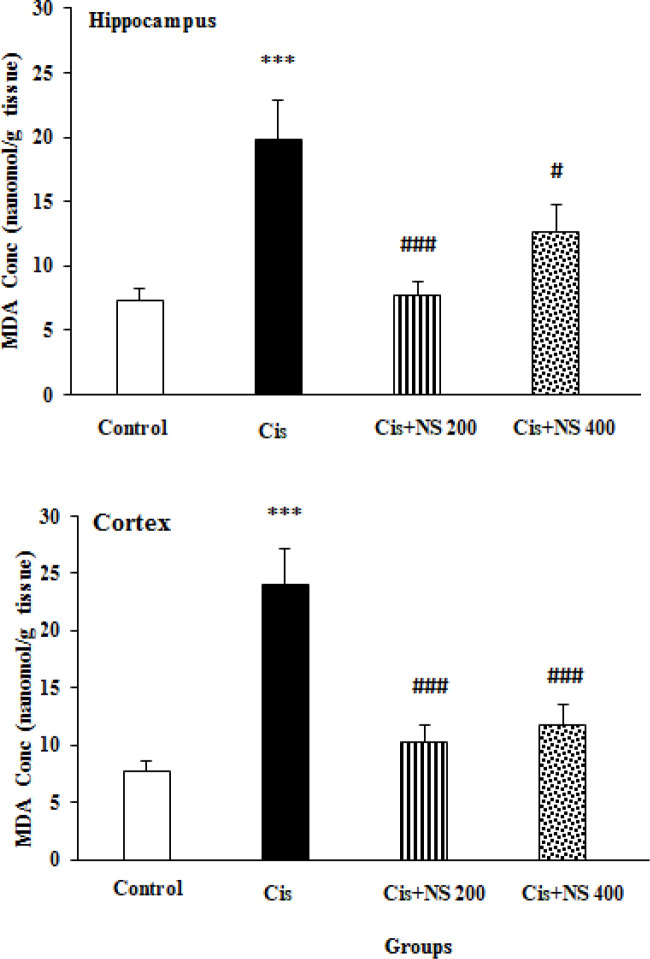
Effects of *Nigella sativa* and cisplatin on MDA concentration of hippocampus and cortex. Data is expressed as mean±SEM; ***p<0.001 compared to the control group; ###p<0.001 and #p<0.05 compared to the Cis group [n=8].

The biochemical analysis of the brain tissues indicated that cisplatin injection significantly increased the MDA level in the hippocampal and cortex of the Cis group compared to the control group (p<0.001, Figure 2). The NS administration significantly reduced the MDA level in the Cis+NS 200 group compared to the Cis group (p<0.001). Furthermore, the brain tissues levels of MDA were extremely lower in the NS 400 group than the Cis group (p<0.05 and p<0.001, Figure 2).

**Effects of cisplatin and NS on the brain concentrations of thiol and SOD**

**Figure 3:**
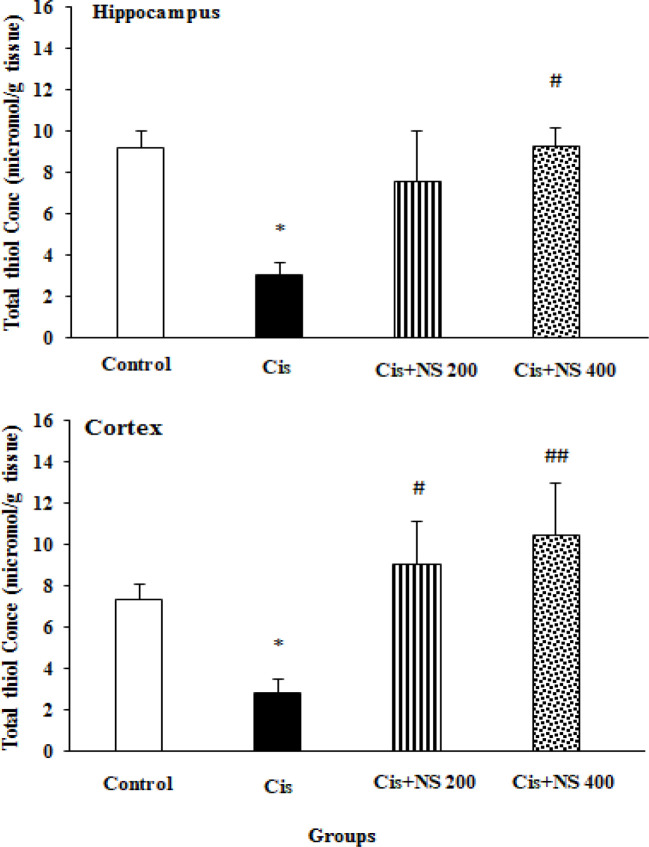
Effects of Nigella sativa and cisplatin on thiol concentration of hippocampus and cortex. Data expressed as mean±SEM; *p<0.05 compared to control group; ###p<0.001, ##p<0.01 and #p<0.05 compared to Cis group [n=8]).

The thiol concentration in both hippocampal and cortex tissues was decreased in the Cis group compared to the control group (p<0.05). The administration of NS (400 mg/kg) significantly enhanced the thiol level in the hippocampal and cortex tissues (p<0.05-p<0.01). In addition, NS treatment (200 mg/kg) enhanced the thiol concentration of the cortex tissue (p<0.05) in the Cis+NS 200 group (Figure 3). 

**Figure 4:**
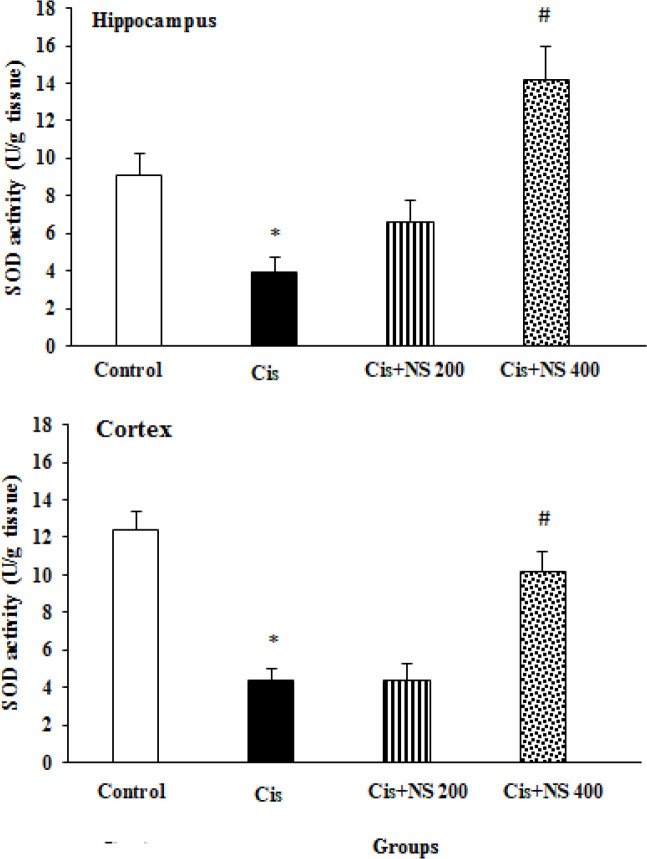
Effects of *Nigella sativa* and cisplatin on SOD concentration of hippocampus and cortex. Data is expressed as mean±SEM; *p<0.05 compared to the control group; #p<0.05 compared to the Cis group [n=8]).

As shown in Figure 4, the injection of cisplatin decreased the SOD activity in the Cis group compared to the control group (p<0.05). Moreover, the administration of NS seed (400 mg/kg) modified the effect of cisplatin by increasing the SOD activity in both hippocampal and cortex tissues of the Cis+NS 400 group compared to the Cis group (p<0.05, Figure 4). The administration of NS extract with dose 200 

mg/kg could not significantly change the effect of cisplatin on SOD activity in the NS and Cis+NS 200 groups (Figure 4).

## Discussion

Neurotoxicity and cognitive impairment are frequently reported during and after cancer treatment (Wefel et al. 2004; Ahles et al., 2007; Vardy et al., 2007). Thus, the main purpose of this study was to investigate the effects of cisplatin and NS on the memory of male Wistar rats. According to the results, the rats were less able to learn and remember after receiving cisplatin, as indicated by their behavior test results. Memory defect was associated with increased MDA, and decreased SOD activity and thiol levels. The administration of the NS seed extract followed by cisplatin treatment reversed the memory deficit and brain biochemical markers. 

In the behavioral test, receiving cisplatin decreased the length of staying in the target quadrant. Previous studies have also shown that the cisplatin-treated animals have less efficiency in attention and memory. (Zhou et al., 2016; Chiu et al., 2017; Chiu et al., 2018). Following cisplatin administration, the induced attention deficit persists for several months (Haider et al., 2014). The memory deficit caused by cisplatin was followed by elevated level of MDA and decreased levels of SOD and thiol. The DNA of cells interacts with cisplatin to exert the cytotoxic effects of cisplatin. Platinum binds to nuclear DNA, leading to cell damage and death. Some studies have suggested that cisplatin toxicity occurs due to producing the oxygen free radicals, and subsequently, it causes excessive oxidation of lipids resulting in changing the physical properties of cellular membranes (Lu et al., 2006). Furthermore, some studies have indicated that oxidative stress causes acute renal toxicity (Nematbakhsh et al., 2012).

Regarding the liver, cisplatin has been reported to damage hepatocyte membranes and increase lipid peroxidation, liver enzyme levels, and cell leakage (Souza et al., 2016). It has also been shown that the imbalance of oxidative status in the brain causes memory impairment (Chen et al., 2019). Considering the central nervous system, previous studies have shown that cisplatin may cross the blood-brain barrier (BBB) (Abou-Elghait et al., 2010). Cisplatin may also bind to proteins which protect the BBB, and cause neurotoxicity (Hassan et al., 2013). Furthermore, cisplatin accumulates in nervous system cells, producing free radicals and damaging cells, which could cause severe neurological damage (Souza et al., 2016). In this research, the administration of NS for two weeks could improve the memory and decrease lipid peroxidation, as approved by the low MDA concentration in the brain. As a result, antioxidant enzymes like SOD increased significantly. Hosseini and colleagues demonstrated that injection of NS prevented depression-like behaviors (Hosseini et al., 2015). In another study, NS noticeably decreased the degeneration of neurons after trauma (Javanbakht et al., 2013). NS administration protected nerve cells against neurotoxicity and cytotoxicity induced by Alzheimer's disease (Alhebshi et al., 2013; Jalali et al., 2009). The ameliorative effects of NS treatment on memory and cognition are caused by the antioxidant property of NS (Kanter et al., 2006; Saleh et al., 2019). The interaction of NS with acetylcholine and glutamate has also been suggested (Jukic et al., 2007). Several studies reported the preservative influence of NS against the toxicities such as nephrotoxicity and hepatotoxicity due to diverse oxidative stress agents (Uz et al., 2008; Yaman et al., 2010; Salama et al., 2011; Elkhateeb et al., 2015; Farooqui et al., 2017; Nader et al., 2010). The neuroprotective effects of NS in experimental studies have also been linked to its antioxidant efficacy (Aboul Ezz et al., 2011; Saleh et al., 2019)

A limitation of this study is that the content of cisplatin in the hippocampus and cortex was not measured. In addition, acetylcholinesterase activity was not evaluated in the brain. Observing the above two factors may give a better understanding of how cisplatin and *Nigella sativa* influence memory and learning.

According to the results, the protective effects of NS against cisplatin-induced oxidative stress in hippocampal and cortex tissues can be related to the beneficial effects of NS on cognitive function of cisplatin-induced memory impairment in male rats.

## Conflicts of interest

The authors have declared that there is no conflict of interest.
